# Ancestral diet transgenerationally influences offspring in a parent-of-origin and sex-specific manner

**DOI:** 10.1098/rstb.2018.0181

**Published:** 2019-01-28

**Authors:** Carmen Emborski, Alexander S. Mikheyev

**Affiliations:** 1 The Institute of Environmental and Human Health, Texas Tech University, Lubbock, TX 79416, USA; 2 Okinawa Institute of Science and Technology, 1919-1 Tancha, Onna, Kunigami District, Okinawa Prefecture 904-0495, Japan; 3 Research School of Biology, Australia National University, 134 Linnaeus Way, Acton, Australian Capital Territory 2601, Australia

**Keywords:** parent-of-origin effects, sex-specific, maternal, paternal, *Drosophila melanogaster*, phenotypic additivity

## Abstract

Parent-of-origin effects, whereby specific phenotypes are differentially inherited paternally or maternally, provide useful clues to better understand transgenerational effect transmission. Ancestral diet influences offspring phenotypes, including body composition and fitness. However, the specific role that mothers and fathers play in the transmission of altered phenotypes to male and female offspring remains unclear. We investigated the influence of the parent-of-origin's diet on adult progeny phenotypes and reproductive output for three generations in fruit flies (*Drosophila melanogaster*). Males and females reared on a control diet were exposed to the control diet or one of two altered (no- or high-) sugar treatment diets for a single generation. Flies from one of the two altered diet treatments were then mated to control flies in a full-factorial design to produce F_1_ offspring and kept on control media for each following generation. We found parent-of-origin (triglyceride) and non-parent-of-origin (sugar) body composition effects, which were transgenerational and sex-specific. Additionally, we observed a negative correlation between intergenerational maternal reproductive output and triglyceride levels, suggesting that ancestral diet may affect fitness. This work demonstrates that ancestral diet can transmit altered phenotypes in a parent-of-origin and sex-specific manner and highlights that mechanisms regulating such transmission have been greatly overlooked.

This article is part of the theme issue ‘The role of plasticity in phenotypic adaptation to rapid environmental change’.

## 1. Introduction

Ancestral exposures can transgenerationally alter offspring phenotypic expression [1], influencing diverse biological processes ranging from phenotypic plasticity to obesity [2,3]. Parental nutrition is considered particularly important in influencing offspring phenotypes [4]. In humans, alterations in ancestral food resources, such as starvation and overnutrition, have been strongly associated with multigenerational inheritance of diseases such as obesity, diabetes and cardiovascular disease [5,6]. In model organisms, such as mice and fruit flies, dietary changes have been associated with altered body composition, reproductive output, behaviour and immunity [7]. Together, unbalanced and altered diets have repeatedly been shown to influence offspring phenotypes in a wide variety of species, yet our understanding of how and why this transmission occurs remains poorly understood [6].

Parent-of-origin effects, whereby specific phenotypes are differentially inherited either maternally or paternally by offspring, provide useful clues to better understand transgenerational inheritance, and represent a first step to home in on possible modes of transmission [8,9]. Maternal effects have long been recognized as an important non-genetic source of phenotypic variation in a range of organisms owing to embryonic nutritional provisioning [10,11]. Paternal effects are often assumed to be absent or much less important than maternal effects, particularly in organisms that lack conventional paternal provisioning and care [12]. However, several recent studies suggest that environment-dependent paternal effects can occur in the absence of conventional paternal care [13–16]. Taken together, it has become clear that mothers and fathers both influence their progeny, yet how, why, and the extent of that influence may differ [8]. Additionally, sex-specific offspring responses to ancestrally transmitted cues may differ, which has been shown in many organisms from *Drosophila* to humans to mice [9,14,17–21]. This is particularly important given that sex can account for as much as 45% of the variance observed in offspring phenotypic responses to ancestral environments [21]. Thus, when determining the influence of cross-generational effects, considering the relative importance of each parent-of-origin on male and female offspring responses and reproductive output may provide useful clues to better understand ancestral influence on offspring phenotypes.

A growing number of studies have analysed maternal and paternal dietary influences on sex-specific offspring phenotypes, most of which analyse over intergenerational timeframes [13,14,18,22–25]. Although intergenerational studies may provide some insight into understanding modes of transmission across generations, they are confounded by the presence and direct exposure of the germ cell in the parent [26]. Transgenerational studies remove this confounding factor because effects on offspring are analysed beyond the generation(s) of direct exposure [26]. To date, only a small number of studies have experimentally analysed sex-specific maternal and paternal dietary influences over transgenerational timeframes [21,27,28]. Notably for each of these studies, the combined effect of both parents' exposure is not included in the study design, making it unclear whether the maternal and paternal effects seen are equivalent to the combined effect of both parents.

Additionally, owing to the increasing prevalence of non-communicable disease such as metabolic disease (e.g. diabetes and obesity), there has been significant interest in the influence of ancestral diet on progeny metabolism or body composition [2,16,25,29–35]. However, while terms like ‘obesity’ and ‘metabolic syndrome’ are frequently used in many experimental transgenerational health-focused studies, little work has been done to specifically define measurable parameters of disease onset based on evidence in the used model organisms. For example, although body composition provides a snapshot into an individual's current state, these measurements alone do not provide context of whether these alterations significantly affect an organism's survival or reproductive abilities. Female lifetime reproductive output has a well-documented relationship with body composition [34,36,37], and may help provide better context about whether the observed body composition effects are deleterious, like those seen in obese phenotypes in humans.

With these considerations in mind, we built upon a previous work, both in our laboratory [38] and elsewhere [16,30] that found significant transgenerational effects in body composition phenotypes and fitness in *Drosophila melanogaster* following a single generation exposure to varying sugar diets. In the current study, we tested the transgenerational maternal, paternal and parental effects of an altered sugar diet on the phenotypic responses of male and female offspring and lifetime female reproductive output responses in the fruit fly. Fruit flies have many broad metabolic, digestive and regulatory similarities to mammals and other eukaryotes, allowing for generalizable insights [39,40]. They have the major advantage of short reproductive times, making them easy to study evolutionary relevant endpoints like lifetime reproductive output, in addition to their other biologically relevant endpoints, like body composition. Furthermore, *D. melanogaster* is one species where fathers make no obvious material contribution of offspring [41] and mothers provide little material contribution following oviposition, thus results in the next generation are less confounded by the external influence of parental provisioning and care.

In the current study, we found significant changes in sugar concentrations in fly offspring deriving from both treatments that were sex-specific, but did not appear to derive from a specific parent-of-origin. Additionally, we found both intergenerational and transgenerational triglyceride effects that were sex- and parent-of-origin-specific, where triglyceride levels were maternally altered in male offspring and paternally altered in female offspring. Furthermore, high levels of triglycerides were correlated with intergenerationally decreased maternal reproductive output, suggesting possible effects on health and fitness. Taken together, our work provides phenotypic clues for future mechanistic research, and highlights that environmental and ancestral cues can influence males and females differently, both in transmission and response.

## 2. Material and methods

### (a) Fly stocks

Wild-type (Canton-S) *Drosophila melanogaster* were obtained from Drosophila Genetic Research Center (Kyoto DGRC), Japan. This is strain was maintained in continuous laboratory culture for a century, and individual flies should be genetically homogeneous. Stock flies were raised and maintained in glass vials in a standard yeast/glucose diet (4% yeast, 8% dextrose, 1% agar, 0.4% propionic acid, 0.3% butyl *p*-hydroxybenzonate) at 25°C and 60% relative humidity under 13 L : 11 D light : dark cycles. Prior to this study, flies were maintained with a control diet for more than 35 generations.

### (b) Exposure diets and experimental design

**Figure 1.**
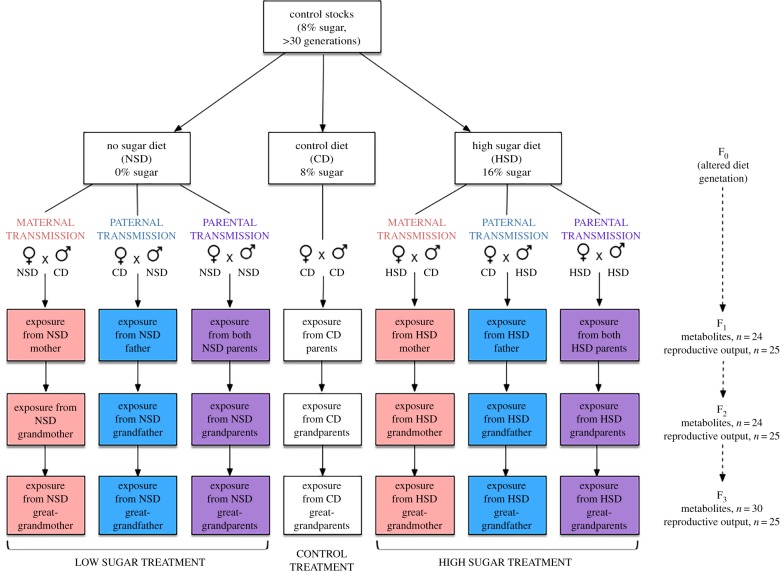
Experimental design. To assess the relative inter- and trans-generational influence of maternal and paternal diet on body composition and reproductive output of descendants, mothers and fathers were challenged with a control diet (CD) or a treatment (high sugar (HSD) or no sugar (NSD)) diet for a single generation and assigned to one of three lines of transmission (i.e. one F_0_ parent, both, or neither were assigned to a given diet). To limit any phenotypic parent-of-origin differences to ancestral diet, F_1_–F_3_ descendants were exposed exclusively to CD media. Body composition (metabolites) was measured in F_1_–F_3_ male and female descendants. Metabolite sample sizes represent the number samples for each sex, parent-of-origin and metabolite within that generation, where each sample contains four pooled subsamples of flies. Reproductive output sample sizes represent the number of single females mated and analysed from each parent-of-origin for each generation, where successful eclosion of offspring was counted throughout the entire life of the fly.

In the first generation (F_0_) of this study, wild-type stock flies were exposed to one of three diets from oviposition to death: no-sugar diet (0% sugar, NSD), control diet (8% sugar, CD) and high-sugar diet (16% sugar, HSD), where all other media ingredients except sugar stayed constant (1% agar, 4% yeast, 0.7% preservative, RO water). Immediately following eclosion, flies (F_0_) were moved to new vials containing the same treatment media that they were reared in until they were 6 days old. Six-day-old F_0_ flies were then transferred to CD media, where each vial contained six females and four males, which corresponded to the intended parent of transmission for each treatment (figure 1). For each subsequent generation, flies continued to be mated according to their parent-of-origin lineage (e.g. maternally transmitted F_1_ flies were mated with six females from a given treatment and four males from control, paternally transmitted flies were mated with six females from controls and four males from the given paternal treatment, and parentally transmitted flies were mated with six parentally transmitted mothers and six parentally transmitted fathers). Flies used to mate the F_1_ generation remained in the CD for 3 days in order to deposit eggs, at which time they were removed and euthanized. Eclosed F_1_ flies were then used for metabolite or reproductive output analysis, or were mated to create the F_2_ generation. Similarly, eclosed F_2_ flies were either used for metabolite or reproductive output analysis, or were mated to create the F_3_ generation. For each generation, treatment and parent-of-origin, flies were mated with non-siblings, where males and females derived from separate vials. Notably, for each of the F_1_–F_3_ generations, each treatment and parent-of-origin group was exposed exclusively to CD media from oviposition to death and all analyses and matings for each generation were done simultaneously (figure 1); thus, any resulting phenotypic between-group differences for a given generation resulted from ancestral and parent of origin exposure differences. Additionally, the density of flies grown in each vial for all generations were controlled by mating six females with four males for 72 h, which was determined as the optimum mating strategy for our targeted population size prior to experimentation.

### (c) Sample collection for metabolite analysis

As the F_0_ generation responses had previously been analysed in two prior studies, which produced consistent results in both previous studies, only the filial 1–3 generations (F_1_–F_3_) were analysed. For all F_1_–F_3_ generations, virgin flies were collected within 8 h of eclosion and stored in sex-separated vials containing fresh CD media. Notably, to prevent pseudoreplication, each pooled sample used for metabolite analysis was maintained in its own vial separate from other samples throughout their life. At 7 days old, these offspring were starved for 24 h in order to clear guts of biasing media contents. After 24 h of starvation, pooled samples of four flies were weighed to the nearest 0.1 mg and processed for metabolite measurements. For metabolites, sample sizes for each generation were as follows: F_1_ (*n* = 24 pooled samples of four flies per sample), F_2_ (*n* = 24 pooled samples of four flies per sample), F_3_ (*n* = 30 pooled samples of four flies per sample).

### (d) Sugar quantification

Pooled whole fly samples were homogenized in ice-cold acetate buffer (pH 5.6), incubated at 95°C for 20 min to prevent degradation, and centrifuged at 12 000 r.p.m. for 2 min. The resulting supernatant was collected for glucose, trehalose and glycogen analysis. Trehalose and glycogen samples were treated with trehalase (0.25 units ml^−1^) and amyloglucosidase (5 units ml^−1^), and incubated for 12 h at 37°C and 60°C, respectively. Resulting glucose levels for three sugars were analysed using Glucose Assay Reagent (Sigma GAHK20), where samples and standards were randomized on the plate(s). For each generation, standards for each sugar were freshly made via serial dilution of a concentrated stock. To determine individual sample concentrations, each sugar's absorbance was first compared to the sugar-specific standard curve. As all three sugars were enzymatically broken down to glucose, as per the method of the assay, the glucose concentration for each sample was subtracted out from each corresponding sample's trehalose and glycogen concentrations. Notably, samples were normalized to weight [42,43].

### (e) Lipid quantification

#### (i) Extraction

Pooled samples were homogenized in 200 µl ice-cold methanol containing internal standards using a Physcotron Handy Micro Homogenizer. Internal standards contained triheptadecanoin, a heavy triglyceride compound not found in nature (Larodan Fine Chemicals). Following homogenization, 400 µl methyl-tert-butyl ether (MTBE) was added to each sample and samples were shaken for 7 min at 1100 r.p.m. Next, 100 µl HPLC-grade H_2_O was added and samples were shaken at 4°C for 30 s at 1000 r.p.m. Samples were then centrifuged at 2000 r.p.m. for 5 min. Finally, 200 µl of the top layer (MTBE containing lipids) was transferred to a new glass insert, speed vacuumed to dryness, and stored at −20°C until analysis.

#### (ii) Analysis and quantification of lipids using UHPLC-MS

For analysis, dried samples were resuspended in 150 µl of toluene and sonicated for 10 min. Then, 10 µl of resuspended sample was added into 90 µl methanol, creating a 10-fold dilution, which was sonicated for 10 min. This resuspension procedure was automated using a PAL Combi-xt autosampler. The autosampler syringe was washed with 400 µl toluene and 200 µl methanol between samples. For each sample, 3 µl of the 10-fold dilution was injected into a Waters ACQUITY UPLC Class-I in tandem with a Waters SYNAPT G2-S high definition mass spectrometer equipped with ion mobility. Lipids were separated in an ACQUITY UPLC CSH C18 1.7 µm 2.1 × 100 mm analytical column at 400 µl min^−1^, 60°C. A separation gradient was used to separate compounds and comprised of two solvents (A and B). Solvent A was comprised of a 60 : 40 acetonitrile : distilled water (10 mM ammonium formate + 0.1% formic acid) solution, and solvent B was comprised of a 90 : 10 2-isopropanol : acetonitrile (10 mM ammonium formate + 0.1% formic acid) solution. The gradient shift began with 85% solvent A and 15% solvent B, shifting to 40% solvent A and 60% solvent B in 3 min, then to 28% solvent A and 72% solvent B in 0.5 min, then to 20% solvent A and 80% solvent B in 4.5 min, then to 0% solvent A and 100% solvent B in 1 min, and held at 99% solvent B for 2 min. The column was then equilibrated for 1 min at 15% solvent B, followed by a post-separation washing gradient of 99% solvent B for 2 min, and a final equilibration at 15% solvent B for 2 min. Total run time was 17 min. Autosampler solvents were comprised of 60 : 40 acetonitrile : distilled water, which was used for aspirating and loading sample into the sample loop, and 90 : 10 2-isopropanol : acetonitrile (0.1% formic acid) for washing the needle to avoid carryover between samples. Mass spectrometer used a LockMass solution of leucine/enkephalin 2 pmol ml^−1^ in 50% acetonitrile (0.1% formic acid) infused every 30 s for automatic mass correction during acquisition time. Mass spectrometer settings were as follows: 2.0 kV spray voltage, cone voltage 30 V, desolvation temperature 400°C, desolvation gas 900 l h^−1^, source temperature 120°C, acquisition range from 50 to 1700 *m/z*, scan rate 10 hz, acquisition mode MSe (independent data acquisition), high resolution 35 000 FWHM, continuum mode, quad profile automatic, collision energy was 6 V for low energy (collision trap), and ramped from 20 to 40 V in high energy mode. Mass spectrometer was calibrated with sodium formate 500 mM in water.

Acquisition of mass spectrometric data was done using Waters MassLynx v4.1. Chromatographic data were processed using MzMine2 open-source software, for mass correction (using acquired lock mass data), alignment, normalization, deconvolution of high energy data (MSe), isotope grouping, peak picking and peak identification based on high energy fragmentation using Lipid Maps database (18 Mar 2014 version). Following peak identification, possible metabolic species were listed and individual compounds were manually assigned from this list based on isotope similarity, compound score (as provided by software), and expected retention times. The total sum of all identified triglycerides was then divided into an internal standard, which was added to the sample prior to processing and provided relative lipid concentrations for each sample.

### (f) Female lifetime reproductive output

Reproductive output represented the total number of successfully eclosed offspring produced by a single female deriving from a particular treatment or control lineage. The number of successfully eclosed offspring were counted from eclosion until death of the female (*n* = 25 for each treatment and generation). Briefly, upon eclosion, one female deriving from an ancestral HSD or NSD parent-of-origin was placed in a vial containing control media with one non-sibling male deriving from CD ancestry (figure 1). To make sure that female reproduction was not limited by male quality, a new male was transferred into each vial every second week, or immediately if escaped during handling or found dead. Flies used to quantify reproductive output were moved to new vials twice per week in order to prevent overcrowding and to reduce counting errors. Twice per week, the number of eclosed flies were counted from each vial and tallied over the course of the female's lifetime.

### (g) Statistical analyses

Data were analysed using R statistical software (version 3.5.0). Linear regressions were used to calculate residuals for the multivariate model, where fixed variables comprised of treatment (i.e. NSD, CD or HSD), parent-of-origin (i.e. maternal, paternal or parental exposure), sex (i.e. male or female), generation (i.e. F_1_, F_2_ or F_3_) and total pooled fly weight (i.e. weight of four flies per sample). We tested two hypotheses. We first analysed whether either treatment (i.e. NSD or HSD) significantly altered metabolite and reproductive output responses relative to controls over intergenerational and transgenerational time. Specifically, we tested the null hypothesis that flies deriving from a given treatment and parent-of-origin did not differ from controls for each sex and generation (e.g. F_1_ CD females = F_1_ NSD parental females). We then analysed whether maternal and paternal effects are additive to the combined effects of both parents. Specifically, we tested the null hypothesis that parentally transmitted responses were equal to the combined interaction of maternally and paternally transmitted responses for a given treatment, generation and sex (e.g. F_1_ NSD parental females = F_1_ NSD maternal × paternal females). For both hypotheses, planned linear contrasts were used to test for significant relationships between variables within the model. Prior to analyses, linear model assumptions were checked. Additionally, in order to account for type I errors associated with multiple comparisons, false discovery rate (FDR) corrections were conducted using the Benjamini–Hochberg procedures [44] to control experiment-wise error rates. All statistics and tables can be found at: https://github.com/cemborski/Parent-of-Origin-Effects-on-Transgenerational-Inheritance-in-Drosophila-melanogaster.

## 3. Results

### (a) Sugar phenotypes display transgenerational sex-specific, but not parent-of-origin-specific effects

**Figure 2.**
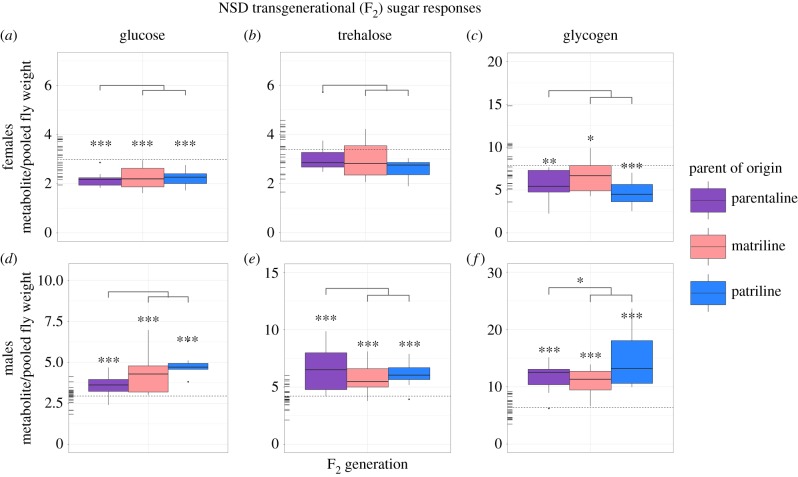
Transgenerational (F_2_) lineage-specific effects of ancestral NSD on sugar phenotypes. Data show raw sugar values for females (*a*–*c*) and males (*d*–*f*). Rug plots located on the left of each plot represent control values for each metabolite in the F_2_ generation, where the mean control value is indicated by the horizontal dotted line. Significance levels were corrected experiment-wide using false discovery rate corrections [44] and are represented by *** (0.001), ** (0.01), * (0.05). Notably, significant differences noted directly above individual boxplots denote differences between controls and a given lineage, whereas significance levels noted in the lines above all three of the lineages denote phenotypic non-additivity between parentally transmitted responses and the sum of maternally and paternally transmitted responses.

**Figure 3.**
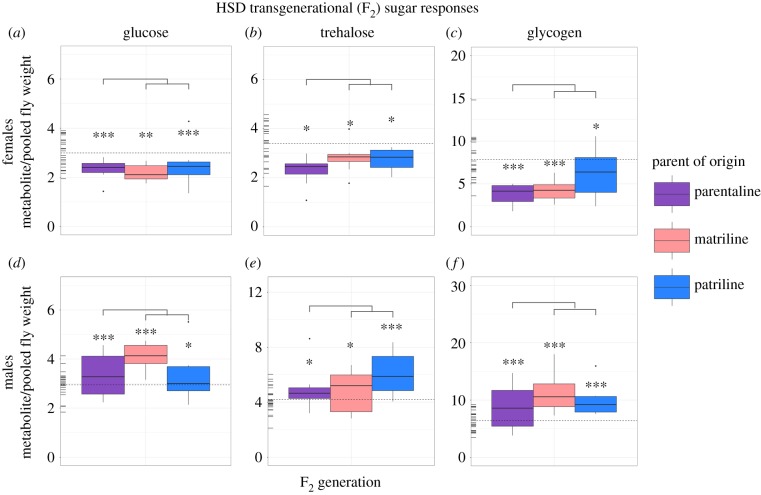
Transgenerational (F_2_) lineage-specific effects of ancestral HSD on sugar phenotypes. Data show raw sugar values for females (*a–c*) and males (*d–f*). Rug plots located on the left of each plot represent control values for each metabolite in the F_2_ generation, where the mean control value is indicated by the horizontal dotted line. Significance levels were corrected experiment wide using false discovery rate corrections [44] and are represented by *** (0.001), ** (0.01), * (0.05). Notably, significant differences noted directly above individual boxplots denote differences between controls and a given lineage, whereas significance levels noted in the lines above all three of the lineages denote phenotypic non-additivity between parentally transmitted responses and the sum of maternally and paternally transmitted responses.

Male and female offspring displayed transgenerationally altered sugar phenotypes in all three parents-of-origin relative to controls in both NSD and HSD treatments. Altered responses were primarily observed in the F_2_ generation (figures 2 and 3), and not in the F_1_ or F_3_ generations (see the github repository identified in the Data accessibility section, figures S1 and S2). With the exception of NSD female trehalose concentrations, all metabolites significantly differed from controls in the F_2_ generation for all three parents-of-origin (i.e. maternal, paternal and parental) (figures 2 and 3; github repository, table S1).

To determine if the maternal and paternally transmitted effects could be additive, we tested whether the sum of maternally and paternally transmitted responses was equivalent to parentally transmitted responses. Generally, significant differences were not detected in sugar phenotypic responses (github repository, table S1), suggesting that parental effects are equal to the sum of their maternal and paternal contributions. This response is observed with the exception of the NSD F_2_ male glycogen concentrations (generalized linear model (GLM), *z* = −3.30, d.f. = 183, *p* = 0.02).

In both NSD and HSD flies, sex-specific effects were observed, where male and female responses significantly differed across all sugar phenotypes (LM, *t*_1089_ = 3.05, *p* = 0.002). Specifically, sugar concentrations in NSD and HSD males were generally significantly higher than control males and sugar concentrations in NSD and HSD females were generally significantly lower than control females (figures 2 and 3; github repository, table S1).

### (b) Triglyceride phenotypes display transgenerational sex-specific, parent-of-origin effects

**Figure 4.**
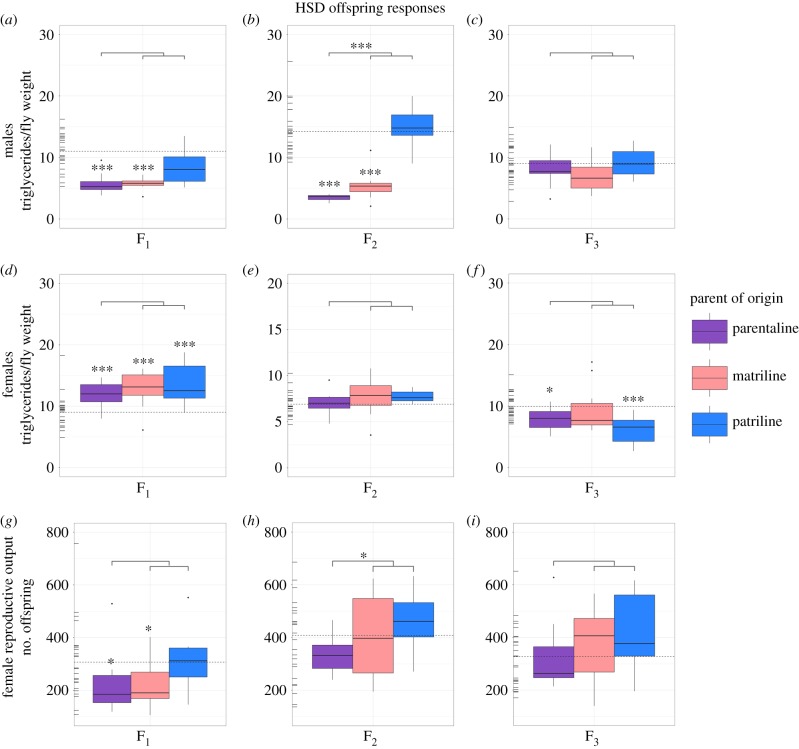
Lineage-specific effects of ancestral HSD on male and female triglyceride levels and female reproductive output. Data show raw values of triglyceride levels in males (*a–c*) and females (*d–f*), as well as total counts of successfully eclosed offspring (*g–i*) of females deriving from an ancestral HSD parent-of-origin for three generations. Rug plots located on the left of each plot represent control values for each metabolite and generation displayed, where the mean control value is indicated by the horizontal dotted line. Significance levels were corrected experiment wide using false discovery rate corrections [44] and are represented by *** (0.001), ** (0.01), * (0.05). Notably, significant differences noted directly above individual boxplots denote differences between controls and a given lineage, whereas significance levels noted in the line above all three of the lineages denote phenotypic non-additivity between parentally transmitted responses and the sum of maternally and paternally transmitted responses.

Sex-specific parent-of-origin effects were sometimes detected in whole body triglyceride levels in response to ancestral HSD. When observed, male triglyceride levels were more strongly influenced by the ancestral maternal exposure (figure 4*a*,*b*) and the female triglyceride levels were more strongly influenced by ancestral paternal exposure (figure 4*f*).

In HSD male triglycerides, responses significantly differed from controls when maternally or parentally transmitted in both the F_1_ and F_2_ generations (GLM, F_1_: (maternal: *t*_183_ = −4.86, *p* ≥ 0.001; parental: *t*_183_ = −5.55, *p* ≥ 0.001), F_2_: (maternal: *t*_183_ = −8.21, *p* ≥ 0.001; F_2_ parental: *t*_183_ = −11.81, *p* ≥ 0.001)). Paternally transmitted triglyceride responses did not significantly differ from controls in any generation for HSD males (F_1_: GLM, *t*_183_ = −2.49, *p* = 0.06; F_2_: GLM, *t*_183_ = 2.22, *p* = 0.11; F_3_: GLM, *t*_183_ = 0.74, *p* = 0.84). To determine if these effects may be additive, we tested whether the sum of maternally and paternally transmitted responses were equivalent to parentally transmitted responses. In the F_1_ HSD male offspring, no significant differences were detected (F_1_: GLM contrast, *z* = −2.16, d.f. = 183, *p* = 0.24). In the F_2_ generation, significant differences were detected, suggesting that transmission by each parent was not equal in F_2_ (F_2_: GLM contrast, *z* = −10.245, d.f. = 183, *p* ≤ 0.001). In the F_3_ generation, no significant differences were detected (F_3_: GLM contrast, *z* = −0.82, d.f. = 183, *p* = 0.999).

In HSD female triglycerides, parent-of-origin specific effects were not observed until the F_3_ generation, where altered responses were transmitted paternally. In the F_1_ generation, responses significantly differed from controls for all three parents-of-origin relative to controls (parental: GLM, *t*_183_ = 5.51, *p* ≥ 0.001; maternal: GLM, *t*_183_ = 4.45, *p* ≥ 0.001; paternal: GLM, *t*_183_ = 4.60, *p* ≥ 0.001) (figure 4*d–f*). In F_2_, no significant differences were detected relative to controls. In F_3_, parentally and paternally transmitted responses significantly differed from controls (F_3_ parental: GLM, *t*_183_ = −3.18, *p* = 0.010; paternal: GLM, *t*_183_ = −4.86, *p* ≥ 0.001). When testing whether responses could be transmitted additively, significant differences were not detected between parentally transmitted responses and the sum of maternally- and paternally- transmitted responses for all three generations analysed.

The parent-of-origin specific responses observed in the HSD flies were not observed in the NSD flies. Specifically, NSD female flies did not significantly differ from controls in any of the three parent lines, nor across any of the generations (github repository, figure S3*a*–*c*). In NSD male flies, significantly maternal and paternal effects were observed in the F_1_ generation, where these offspring were significantly higher than controls (maternal: GLM, *t*_183_ = 3.55, *p* = 0.004; paternal: GLM, *t*_183_ = 9.05, *p* ≤ 0.001). In the F_2_ and F_3_ generations, no effects were detected.

### (c) Female reproductive output intergenerationally, but not transgenerationally, altered in response to an ancestral maternal high sugar diet exposure

Female reproductive output was maternally and parentally influenced in F_1_ HSD flies, as compared to controls (maternal: GLM, *t*_96_ = −2.77, *p* = 0.038; parental: GLM, *t*_96_ = −3.02, *p* = 0.021) (figure 4*g*). In the F_2_ and F_3_ generations, significant differences were not detected between treatment and control responses for any parent-of-origin line (figure 4*h–i*). Notably, reproductive output differences were not detected in the NSD treatment.

Lifetime reproductive output provides a health and evolutionary context for the observed phenotypic changes. Specifically, the HSD was chosen to simulate an obesity-stimulating unhealthy diet, with the expectation of lower reproductive output. Given that significant reproductive output effects were observed in the HSD, we tested the relationship between triglycerides and reproductive output in this treatment and found a negative correlation in females (Pearson's product-moment correlation, *t*_217_ = −4.05, *r* = −0.265, *p* ≤ 0.001).

## 4. Discussion

Ancestral exposures influence offspring phenotypes for several generations. Previously, a number of recent studies have observed significant transgenerational effects in body composition and reproductive output phenotypes in *D. melanogaster* following a single generation exposure to altered diets [21,30,38,45]. Studies examining parent-of-origin, sex-specific effects may provide useful clues to better understand transgenerational inheritance, and represent a first step to home in on possible modes of transmission [9,20,46]. In the current study, we investigated the influence of an ancestrally altered maternal, paternal and parental diet on adult progeny body composition phenotypes and reproductive output. Here, the specific parent-of-origin was exposed to an altered sugar diet for a single generation and transmitted effects were measured in unexposed offspring for three subsequent generations. We observed sex-specific, but not parent-of-origin specific, effects in fly sugar phenotypes for both NSD and HSD treatments. Conversely, we observed intergenerational and transgenerational parent-of-origin effects on triglyceride levels in HSD flies that were also sex-specific. Additionally, we observed intergenerational maternal reproductive output effects in the HSD treatment, which were negatively correlated with fly triglyceride levels. Together, these observations provide clues to help future studies home in on possible modes of transmission, which we discuss in further detail below.

The effects observed in fly sugar phenotypes were present and consistent between all three parental lines for both the NSD and HSD treatment groups, indicating no specific parent-of-origin phenotypic sugar effects. However, sex-specific effects were observed in both treatments, where both NSD and HSD males exhibited higher concentrations of sugars and females exhibited lower sugar concentrations relative to controls (figures 2 and 3). The observed sex-specific differences are not surprising given that sex has been shown to account for as much as 45% of the variance observed in offspring responses to ancestral environments in *Drosophila* [21]. Differences between reproductive strategies or in developmental programming between sexes may explain the observed sex-specific responses [47,48]. Additionally, insulin-like growth factor-1 (IGF-1) signalling has received considerable attention for its influence in regulating energy homeostasis, metabolism, and reproduction within an organism's lifetime, and has been shown to differ between males and females [49–52]. Given our findings, it is possible that IGF-1 may be a viable target of transgenerational transmission mechanisms, influencing the different responses to diet observed between sexes and deserves further attention in the future.

Although not observed in sugar phenotypes, parent-of-origin effects were detected in storage fat (i.e. triglyceride) phenotypes from HSD flies, which were also sex-specific. Here, altered triglyceride levels were observed in male offspring deriving from the ancestral (F_0_) HSD exposed mothers and in female offspring deriving from ancestral (F_0_) HSD exposed fathers (figure 4). Generally, the parent-of-origin sex-specific trends observed in our study were broadly consistent with two out of three previous studies that investigated ancestral dietary overnutrition on body composition phenotypes in flies [21,30], where paternal exposure influenced female offspring body composition phenotypes and maternal exposure influenced male offspring body composition phenotypes. In the third study, Ost and colleagues analysed the paternal influence of a short duration (2 day) exposure to an HSD on male offspring, and found intergenerational, but not transgenerational, paternal effects on male triglyceride levels [16]. Although female (i.e. maternal or offspring) effects were not analysed by Ost and colleagues, their findings do not match the overall observed trends in triglyceride concentrations, as our study did not detect paternal triglyceride effects in male offspring. Notably, the response differences observed could be owing to differences in genetic lines used between the two studies [21]. Despite this, Ost and colleagues detected similar chromatin signatures between the sperm of exposed fathers and phenotypically altered sons [16]. In the future, it would be interesting to test if these chromatin modifications are exclusively paternally transmitted and if they influence offspring phenotypes of both sexes equally.

Through cross-generational transmission, offspring may receive information about their ancestor's environment additively from both parents. This is notable, as many transgenerational studies work under the largely implicit assumption that both parents contribute additively to their offspring's phenotypic or transcriptional output [21,27,28,53]. Yet whether complex transgenerational cues are additively integrated into offspring traits remains largely unknown. In the current study maternal, paternal, and the combined parental transmission effects were measured, allowing us to assess potential phenotypic additivity within our experiment. We observed that most phenotypic traits displayed additive phenotypic transmission from both parents, with the exception of F_2_ HSD triglycerides and F_2_ NSD glycogen concentrations in male offspring. Why some phenotypes show non-additive effects is unclear in the current study, but highlights additional levels of complexity in transgenerational inheritance. Research analysing gene transcription and mapping of complex traits such as those seen here may help better elucidate this intriguing occurrence.

To better elucidate how altered ancestral diets may influence overall health and possibly affect fitness, we also measured lifetime female reproductive output. We observed decreased female reproductive output in conjunction with increased triglycerides in F_1_ HSD matriline females, indicating a possible deleterious intergenerational effect of offspring body composition levels (i.e. obese-like phenotype). Notably, female reproductive output was negatively correlated with triglyceride levels. As increased body fat has previously been associated with decreased body fat in a number of epidemiological and laboratory studies, and in a range of animals, this finding is not necessarily surprising [54–56]. Reproduction was not affected in HSD F_2_ and F_3_ descendants from any parent-of-origin line relative to controls, nor in the NSD treatment flies. Notably, the unaffected reproductive output is also correspondingly observed with unchanged or decreased triglyceride levels in female flies. As HSD F_1_ reproductive output effects were observed from ancestral maternal and parental (but not paternal) exposure, it is clear that these effects were maternally transmitted. However, it remains unclear whether these effects are in response to the observed transgenerational body composition effects, a result of alterations in maternal provisioning, or owing to direct offspring exposure effects.

It is worth noting that we only measured reproductive output females, and under near-ideal conditions. Thus, it is possible that males or females under more stressful conditions could show qualitatively different effects on fitness-related traits, or that other traits, such as mating success may show different responses. It is also important to note that, for many species in the field, access to nutrients may be limiting, in which case fitness may negatively correlate with fat stores [57–59]. Our study aimed at examining effects of extreme diets, and its treatments are not necessarily relevant to field-like conditions. Rather, the highest sugar level treatments may not be nutritional states commonly encountered by wild animals, but could possibly represent obesogenic modern diets encountered by humans and other animals inhabiting human-associated environments [60]. The interplay between ancestral diet, body composition and reproduction deserves further study, particularly in model organisms, as it links the inherited metabolic physiology to an evolutionary relevant measure of health.

To date, the potential mechanisms mediating transgenerational inheritance specific to maternal and/or paternal exposures are still largely unclear [61,62]. Although we did not test for specific mechanisms in the current study, our results may provide future studies useful clues about how phenotypes may or may not be transmitted. For example, given that fly body composition (i.e. sugar and fat) responses go beyond the F_1_ generation in our study, we are able to largely separate transgenerational mediated mechanisms from direct exposure effects in the offspring [26]. This is particularly notable given that a number of recent studies and reviews have attributed intergenerational parent-of-origin sex-specific effects to direct maternal provisioning effects or gamete-specific plasticity [61,63–65]. Additionally, given that the transgenerational sugar phenotypic effects were transmitted through both the maternal and paternal germ lines, it seems unlikely that the results observed here are a result of mitochondrial DNA, as mitochondria are primarily maternally inherited [66]. Furthermore, given the short effect timeframe and the highly inbred line of flies used, it seems unlikely that genetic effects could be mediating the observed responses in triglycerides or sugars, though effects of selection in the F_0_ generation cannot be, strictly speaking, ruled out.

Epigenetic modifications (e.g. DNA methylation, histone modifications and small non-coding RNAs) are strong candidates influencing the observed effects [8,67,68], particularly given the short timescale observed between exposure and cross-generational effects without the influence of an obvious genetic bottleneck. For example, genomic imprinting is a commonly used epigenetic explanation for parent-of-origin effects in the literature, to the point that the term parent-of-origin is often used synonymously with genomic imprinting. Genomic imprinting is an epigenetic process that marks chromatin in a sex-dependent manner, essentially escaping the epigenetic reprogramming events following fertilization, resulting in differential parent-of-origin gene expression [69]. Given this, genomic imprinting appears to be a plausible explanation of the observed triglyceride responses. In flies, however, the presence of genomic imprinting is controversial owing to findings of alternative explanations for some parent-of-origin effects, the low levels of genome-wide DNA methylation found, as well as because both gynogenetic and androgenetic offspring are viable and fertile in *Drosophila* [70–77]. Additionally, it is still unclear to what degree environmental perturbations effect imprinted marks [20,78]. However, as flies have the machinery necessary for imprinting (i.e. DNA methyltransferase) and a small amount of DNA methylation and extensive chromatin markings have been detected [69,73,79,80], it is possible that imprinting may influence the triglyceride responses observed in this study.

Alternative transmission mechanisms beyond the commonly cited epigenetic mechanisms could also influence the transmission of altered phenotypes across generations. For example, the influence of transgenerational maternal provisioning to the egg has been documented in a number of species, from mammals to birds to insects [81–83]. In insects, a recent study looking at the common house cricket (*Acheta domesticus*) found that mothers could provide variable amounts of active ecdysteroid hormones to their eggs across transgenerational timescales, which was based on the quality of nutrition available to the maternal grandmother [82]. Although less commonly considered, paternal contributions may also influence progeny phenotypes, even in organisms that lack direct paternal provisioning and care. For example, in *Drosophila,* changes in male seminal fluid can alter female postcopulatory behaviours, including feeding behaviour [84], which could have longer cross-generational effects. Although it is unclear the extent to which seminal fluid contents are influenced by diet or metabolic phenotype [85], such interactions could have potential transgenerational implications. In the future, it would be interesting to investigate the influence that seminal fluid has in transgenerational inheritance, as well as whether parental provisioning can lead to the sex-specific trends observed in the current study.

## 5. Conclusion

In conclusion, we show that ancestral dietary alterations can influence progeny plasticity in a sex- and parent-of-origin-specific manner. In combination with previous studies, it is increasingly clear that both the sex of the ancestor that experienced the event and the sex of the individual that receives the information matters. In our system, we show a link between metabolic physiology and a measure of female health. This study highlights the need for further investigation of the interplay between ancestral diet, body composition and reproduction in order to better define evidence-based measurable parameters of disease onset in model organisms. This is particularly true because combined parental effects may be non-additive, introducing additional complexity. Although mechanisms were not analysed in the current study, this work provides phenotypic clues for future research analysing the mechanistic underpinnings of transgenerational effects. From this, we highlight the need for additional parent-of-origin phenotypic and mechanistic studies in a range of the other organisms to better define the roles that mothers and fathers play in, and the functional significance of, transgenerational phenotypic effects.

## Data accessibility

All data, statistics and tables analysed for this publication can accessed via the github repository at: https://github.com/cemborski/Parent-of-Origin-Effects-on-Transgenerational-Inheritance-in-Drosophila-melanogaster.
